# Lateral visual occlusion does not change walking trajectories

**DOI:** 10.1167/18.9.11

**Published:** 2018-09-11

**Authors:** Matt J. Dunn, Simon K. Rushton

**Affiliations:** RushtonSK@cardiff.ac.uk http://psych.cf.ac.uk/contactsandpeople/rushtonsk.php; School of Optometry and Vision Sciences, Cardiff University, Cardiff, UK; School of Psychology, Cardiff University, Cardiff, UK

**Keywords:** *walking*, *hemianopia*, *visual neglect*, *optic flow*, *virtual reality*

## Abstract

Difficulties with walking are often reported following brain damage that causes a lateralized loss of awareness on one side. Whether lateralized loss of awareness has a direct causal impact on walking is unknown. A review of the literature on visually guided walking suggests several reasons why a lateralized loss of visual awareness might be expected to lead to difficulties walking. Here, we isolated and examined the effect of lateralized vision loss on walking behavior in real and virtual environments. Healthy young participants walked to a target placed within a real room, in a virtual corridor, or on a virtual ground plane. In the ground-plane condition, the scene either was empty or contained three obstacles. We reduced vision on one side by occluding one eye (Experiment 1 and 2) or removing one hemifield, defined relative to either the head or trunk (Experiment 2), through use of eye patching (Experiment 1) and a virtual-reality system (Experiment 2). Visual-field restrictions did not induce significant deviations in walking paths in any of the occlusion conditions or any of the environments. The results provide further insight into the visual information that guides walking in humans, and suggest that lateralized vision loss on its own is not the primary cause of walking difficulties.

## Introduction

Following acquired brain injury, it is common for people to encounter difficulties with walking (Carvalho-Pinto & Faria, 2016). In many cases, these difficulties are due to problems generating physical movements or maintaining balance (Langhorne, Coupar, & Pollock, 2009). Other difficulties may have a perceptual origin. There are two common conditions in which the awareness of objects on one side of space is impaired: *homonymous hemianopia* (HH) and *unilateral visual neglect* (UVN). HH, following damage to the optic radiations or primary visual cortex (Hutchins & Corbett, 1997), is the loss of vision in one hemifield, to the left or right of fixation (Millodot, 2004). Detailed reports in the literature of people with HH colliding with objects and having other difficulties walking (M. Warren, 2009) are sparse, but the problem is broadly recognized (Chokron, Perez, & Peyrin, 2016) and solutions have been proposed (e.g., Bowers, Keeney, & Peli, 2008; Pundlik, Tomasi, & Luo, 2015). UVN, which can result from damage to a number of sites (Vallar, 1998; Verdon, Schwartz, Lovblad, Hauert, & Vuilleumier, 2010) but most commonly parietal damage, is not a loss of vision but a loss of awareness of one side of space (Vallar, 1998)—an attentional or representational impairment (Heilman & Valenstein, 2011). People with UVN (many of whom will also have HH) are reported to bump into obstacles and take curved or abnormal trajectories (e.g., Huitema et al., 2006; Robertson, Tegnér, Goodrich, & Wilson, 1994; Tromp, Dinkla, & Mulder, 1995; Turton et al., 2009). Collisions with obstacles have also been reported in people with UVN (Aravind, Darekar, Fung, & Lamontagne, 2015; Turton et al., 2009).

Examining the literature on the visual guidance of locomotion reveals three possible reasons why a lateralized loss of vision or awareness might lead to changes in walking trajectories: a change in perceived direction, a change in perceived heading, or the removal of a strategy for guiding walking.

**Figure 1:**
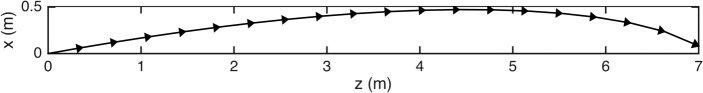
Predicted trajectory (plan view) of an observer who perceives straight-ahead 10° to the right of true straight-ahead, walking from (0, 0) to a target at (7, 0). At each step, the observer orients to place the target perceptually straight-ahead and then takes a step forward. The result is an equiangular trajectory towards the target.

Both HH and UVN may introduce an asymmetry in the functional visual field. Asymmetric visual fields can create a bias in perception of egocentric direction; this is illustrated by a study with monocular patching that found a small but consistent shift in perceived straight-ahead (Porac & Coren, 1986). A shift in perceived straight-ahead has been reported to be associated with HH and UVN (Ferber & Karnath, 1999). HH is associated with a shift toward the blind hemifield, while UVN is associated with a shift away from the neglected hemifield (Ferber & Karnath, 1999). When an observer walks toward a target, they do so by regulating their direction of travel so as to keep the target perceptually straight-ahead (Rushton, Harris, Lloyd, & Wann, 1998). If straight-ahead is perceived accurately, the result will be a straight-line course to the target. If straight-ahead is misperceived, the result will be a curving trajectory; this is illustrated in Figure 1, which shows the predicted trajectory when straight-ahead is perceived 10° to the right. Note that if someone taking a path similar to that shown in Figure 1 believes that they are taking a straight path, they would incorrectly anticipate their future trajectory. Therefore, it would be unsurprising if they bumped into objects (something we have often observed when individuals wear prism glasses that shift the perceived egocentric directions of objects).

Second, a change in perceived heading might be expected to affect walk paths. Observers can judge their direction of travel—their *heading*—from patterns of optic flow (the global patterns of motion picked up at the eye when moving through space; Gibson, 1950; Li & Cheng, 2011). In theory, an asymmetric visual field should not impair the ability to judge heading. The key relation in the optic flow field, the relative position of the target and the optically specified direction of heading, is unchanged (see Rushton et al., 1998). However, Telford and Howard (1996) found that when visual motion was reduced on one side, and the head fixed, observers systematically misperceived their heading direction. Therefore, removal of part of the visual field on one side could bias perceived heading, and if perceived heading is used in the visual guidance of walking (e.g., W. H. Warren, Kay, Zosh, Duchon, & Sahuc, 2001), this would lead to curved walking trajectories similar to those shown in Figure 1. We note that Kountouriotis et al. (2013) have reported that when flow on one side is removed, there is a systematic displacement of the path taken when driving around bends in a simulator.

Third, loss of vision on one side may impair the use of a flow-equalization strategy. Insects control their lateral position when flying through corridors or gaps by keeping the visual flow equal on both sides. This is demonstrated by moving the texture on one side wall (and hence increasing or decreasing the flow rate), which causes the insect to change its lateral position to equalize the flow (Srinivasan & Gregory, 1992). It has been reported that people walking down a *virtual* corridor make compensatory changes in lateral position to equalize flow (Duchon & Warren, 2002). If humans make some use of an equalization strategy, then HH would preclude its use. The result might be an increased variability of lateral position due to the loss of information, or alternatively the adoption of a different strategy such as hugging a wall or path edge.

In summary, three separate lines of theoretical work suggest reasons why a lateralized loss of vision or awareness might be expected to produce changes in walking trajectories, and hence may make some contribution to the difficulties that follow acquired brain injury. Here, we isolate and examine the effect that lateralized visual occlusion can have on walking, by measuring walking trajectories in healthy young individuals during lateralized occlusion of the visual field. We use both simple (eye-patching) and sophisticated (virtual-reality) approaches to remove different parts of the binocular visual field, and we measure walking trajectories in a range of different environments. We test using young healthy participants rather than people with brain injury so that we can remove confounding motor and balance factors.

### Scope of the experiments: Lateralized loss of vision

Simulation of HH is difficult. HH is defined relative to the retina. It is not practical to simulate HH by occluding a retinal hemifield. Although eye trackers have been fitted to head-mounted displays (HMDs; e.g., SMI Eye Tracking HMD Upgrade for the Oculus Rift DK2, SMI, Teltow, Germany), a gaze-contingent display is impractical because bounce and sway of the head while walking are likely to disrupt eye tracking, and the temporal resolution of the eye tracker and HMD are both limited. Simulating HH with contact lenses appears a promising route, but it would be very difficult to maintain the orientation and alignment of such lenses, and due to diffraction it would not be possible to create a clear occlusion edge. Lastly, when HH results from cortical damage, the “blind” field is not occluded; rather, it does not in any meaningful way exist—the primary neural tissue that supports perception of the blind field is missing.

Simulation of UVN is complicated. UVN is a lack of awareness on the neglected side. It is defined primarily relative to the trunk, but it is also influenced by the position of the eye and the head (Mozer, 2002). Additionally, UVN includes an object-based component: One side of each object is neglected (Kerkhoff, 2001). UVN is not normally characterized by a sharply defined edge between the neglected and nonneglected portions of space, but rather as a gradient, albeit a steep one, running from the nonneglected to the neglected side (De Renzi, Gentilini, Faglioni, & Barbieri, 1989; Kinsbourne, 1993). Finally, one of the key features of UVN is a lack of awareness of the space that is neglected.

It is consequently difficult to produce a faithful simulation of either HH or UVN. However, it is not our aim to *simulate* HH and UVN, but rather to ask whether the lateralized loss of the functional visual field that accompanies these conditions has a causal impact on walking. This question can be answered through use of a straightforward occlusion of vision.

We begin with the simplest manipulation: patching one eye. Patching creates an asymmetric field of view—it occludes the peripheral portion of the visual field, the *monocular temporal crescent* on the patched side. Monocular patching has been reported to produce a shift in perceived straight-ahead (Porac & Coren, 1986). It also introduces an asymmetry in the optic flow field, which could impair the functioning of a flow-equalization mechanism or introduce a bias in perceived heading. Any or all of these effects might be expected to produce a change in walking trajectories.

In a second set of experiments, we used virtual-reality equipment (HMD and motion tracker) to occlude one visual hemifield. The hemifield was defined relative to either the head or the trunk. We investigated the effect of different environments (closed corridors vs. open spaces), and because we could create virtual obstacles that did not pose a threat to safety, we also investigated how obstacles affected walk behavior in each condition. The same logic that applies to the first experiment also predicts a change in walking trajectories in this experiment.

## Experiment 1: The effect of monocular patching on walking trajectories

Healthy young participants walked toward a target object with one eye patched. Patching does not remove a full binocular hemifield, but it removes around 30°–40°. For a typical individual, vision toward the patched side is limited to a maximum of 60° (compared to 90°–100° on the unpatched side; Rowe, 2006). To get a feel for how much is occluded, consider an observer facing down the middle of a corridor of 4 m width: The nearest part of the corridor that would be visible on the patched side would be 3.5 m ahead.

While participants walked, their trajectories were monitored with an optical motion-capture system. The motion-capture equipment was mounted on the ceiling of a large rectangular room, and the environment was therefore necessarily limited to an enclosed space. This is potentially important, because rooms and other regular enclosed spaces such as corridors provide a range of perspective and positional cues to the position of the participant relative to the surrounding environment that are not available in natural, irregular, open environments. These cues would likely attenuate, but we hoped would not abolish, the effect of any changes on perceived straight-ahead, heading perception, or flow equalization. We note that although a rectangular room may not be a *natural* environment, it is a typical environment for modern humans.

### Methods

#### Participants

Twelve healthy young adults with no history of neurological trauma were recruited for this study (six female, six male). Informed consent was obtained from the participants after explanation of the nature and possible consequences of the study. The study was conducted in accordance with the Declaration of Helsinki, and under approval given by the School of Psychology Ethics Committee at Cardiff University.

We required for inclusion (see Experiment 2 for further explanation) that participants did not have strabismus or poor stereopsis (>120″ stereoacuity), which was confirmed by an optometrist. All participants recruited were suitable on this basis.

#### Equipment and visual environment

Experiments were conducted in a large carpeted room (8 × 8 m) with no windows. The room was empty but for typical laboratory equipment such as computers, tables, and chairs, located around the edges. Two white floor markers were positioned along one of the room diagonals, 7 m apart.

The position and orientation of the participant's head and trunk were monitored in real time using an optical motion-capture system (Impulse X2, PhaseSpace, San Leandro, CA). Participants wore a cycle helmet and rigid backpack to which were attached LEDs, tracked by 16 cameras mounted on the ceiling of the room. From the position of the LEDs, we were able to infer the position and orientation of the head and trunk. The motion-capture system had a very high sampling frequency (960 Hz) and low latency (<10 ms). The most recent position and orientation data were polled and recorded at 60 Hz.

Walking data were recorded by a laptop PC (Windows 7, Intel i5, NVIDIA GeForce GT 750M) placed within the backpack and linked wirelessly to the motion-capture system. The laptop ran Vizard (WorldViz, Santa Barbara, CA), which simplified data collection from the motion-capture system.

In order to standardize walking speed across trials, participants carried a portable electronic metronome in a pocket.

#### Procedure

To ensure that the participants met our inclusion criteria, an optometrist assessed oculomotor balance for the presence of strabismus under both distance- and near-viewing conditions using a cover test, and measured stereoacuity using the Titmus stereotest (Precision Vision, LaSalle, IL). Interpupillary distance was measured in order to calibrate the images presented within the HMD in the follow-on virtual-reality experiment (see Experiment 2).

Participants were fitted with the helmet and backpack and invited to walk around the laboratory. Once comfortable walking, they were asked to set the metronome to sound at a comfortable walking step frequency. The metronome would play during each experimental trial, and participants were asked to maintain a consistent walking speed across all trials.

Three walks were performed under binocular conditions to establish baseline trajectories, followed by three with the left eye patched. We chose this fixed order because it allowed participants to become comfortable with the experiment before we introduced the eye patch. It also protected against any adaptation effects due to biasing of subsequent baseline measures by patching.

At the beginning of each trial, participants stood near the start marker, facing into the corner. They would then turn and face the target marker, positioned 7 m away on the floor, before walking toward it. They were instructed to stop one step before the marker. Walking direction across the laboratory was the same for each trial, with participants returning to the same start marker before each recorded walk. A response button was carried during the experiment to standardize the protocol with that of Experiment 2, but no response was required in this experiment.

#### Data analyses

Walk-path data were low-pass filtered with a second-order Butterworth filter (cutoff: 0.6 Hz) to minimize the effects of postural sway. As is standard (e.g., W. H. Warren et al., 2001), the first and last part (here, 1 m) of each walk were omitted from analysis because estimates of heading direction are unreliable during these phases. Any gaps in the walk data were interpolated with cubic splines (0.13% of the data were interpolated in this way). Median head position was calculated for each 10-cm segment of each walk. Group mean walk paths were calculated for each walk condition. These analyses were performed using MATLAB (MathWorks, Natick, MA). Descriptive analyses and manipulation of the resulting dataset were performed in the R Environment for Statistical Computing (R Core Team, 2012).

Our key interest is finding whether a lateralized loss of vision could account for the changes in walking trajectories observed in people with brain injury. This interest guides the analysis we report. Effect size, rather than *p*, is used to assess clinical significance. Effect size is a measure of the mean difference between groups (patient/treatment vs. control) compared to the mean difference within groups. Hedges's *g*, the measure of effect size we use here, is (*M*_1_ – *M*_2_)/*SD*, where standard deviation (*SD*) is weighted and pooled. Hedges's *g* is a variant of Cohen's *d* for small samples (Lakens, 2013). An effect size of >2 is a standard cutoff for clinical significance (Jacobson, Follette, & Revenstorf, 1984). Statistical significance can be assessed by adding confidence intervals (CIs) to the measure of effect size (for an introduction to this approach, see Nakagawa & Cuthill, 2007). For example, an effect size of 3 with a 95% CI [−0.4, 6.2] is clinically significant but falls short of statistical significance (because 0 is included within the CI), whereas an effect size of 0.9 with a 95% CI [0.4, 1.4] is not clinically significant but is statistically significant. We calculate 95% CIs for Hedges's *g* using the method described by Cumming and Finch (2001).

We use analysis of variance (ANOVA) to test for order effects before combining data, and to investigate differences between environments. We also use ANOVA in the analysis of head orientation.

### Results and discussion

**Figure 2:**
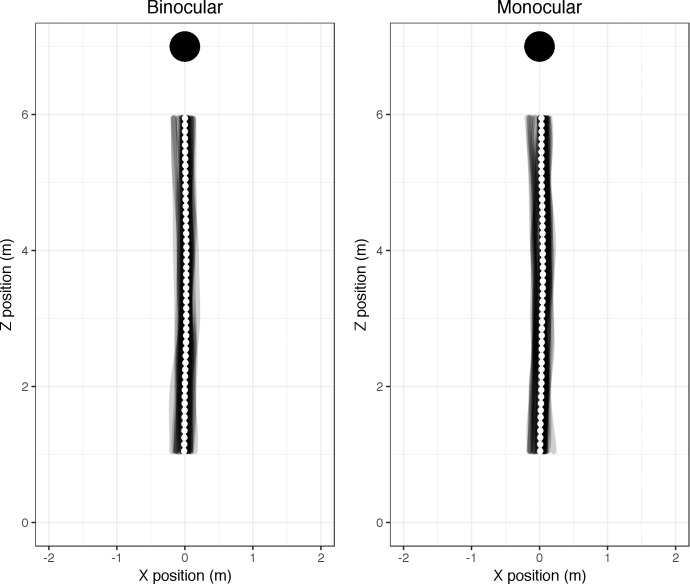
Walk trajectories in plan view under binocular and monocular viewing conditions. Participants started at (0, 0) and walked toward the target at (0, 7). Individual recordings are shown in gray, with the mean walk path (with error bars indicating standard error) overlaid in white for each 10-cm segment. Note that the error bars cannot be distinguished here, because the standard error is smaller than the width of the marker.

Figure 2 shows the mean trajectories (over participant and over trials) in the binocular (baseline) and monocular conditions. Inspection of Figure 2 indicates no obvious difference between the two conditions. We calculated the mean target-heading angle for each participant for each trial for both the monocular and binocular conditions.

We first checked for evidence of adaptation (a difference in walking trajectories over trials), as adaptation would complicate interpretation of the data. We performed a 2 × 3 repeated-measures ANOVA and found no evidence of adaptation across trials—main effect of trial: *F*(2, 20) = 1.81, *η*_p_^2^ = 0.15, *p* = 0.19—or of an interaction between trial and condition, *F*(2, 20) = 1.60, *η*_p_^2^ = 0.14, *p* = 0.23. Because of this, we collapsed the data over trial.

Target-heading angle was, on average, 0.46° to the right across all monocular trials, relative to trials with binocular viewing. Hedges's *g* was 0.55 (95% CI [−0.31, 1.41]).

To place the results in perspective: Although the difference between binocular and monocular viewing is not clinically or statistically significant, the direction and magnitude of the shift is similar to the ∼0.35° shift due to eye patching reported by Porac and Coren (1986). The shift is considerably less than the magnitude of the error in perceived heading (≥10°) reported by Telford and Howard (1996). The similarity in variability in the two conditions (standard deviation in target-heading angle = 0.46° across all binocular trials; 0.50° in the monocular trials) is not in line with the loss of an important flow-equalization strategy in the monocular condition.

One consideration is whether there were factors specific to this experiment that might have attenuated any effect of lateralized visual occlusion on walking trajectories. First, as already noted, the room environment was rich with positional and geometric cues that could have guided straight walking trajectories. For example, the change of perspective shape of the interior corners of a room provides a potentially strong cue to walking direction (Beusmans, 1998). Second, the restriction of the visual field was only partial. By occluding an eye, we produce an asymmetric visual field, but the participant can still see some objects on the occluded side using the unpatched eye. Third, we placed the target on the ground. Although people walk to targets on the ground, it is more typical to fixate objects above the ground, for example when walking to a doorway or a friend. By placing the target on the ground we magnified the optic flow, which may have led to a straightening of the paths (see Harris & Carré, 2001). Lastly, in this experiment the participants may have been overly careful to walk a straight path. They had no other task to perform, and therefore may have paid an atypical amount of attention to their path.

In a second experiment, we switched to using virtual environments, which allowed us to address some of the potential limitations of the first study, add some features, and test the generality of the findings of the first experiment.

## Experiment 2: Partially occluded virtual environments, trajectories, and obstacles

**Figure 3:**
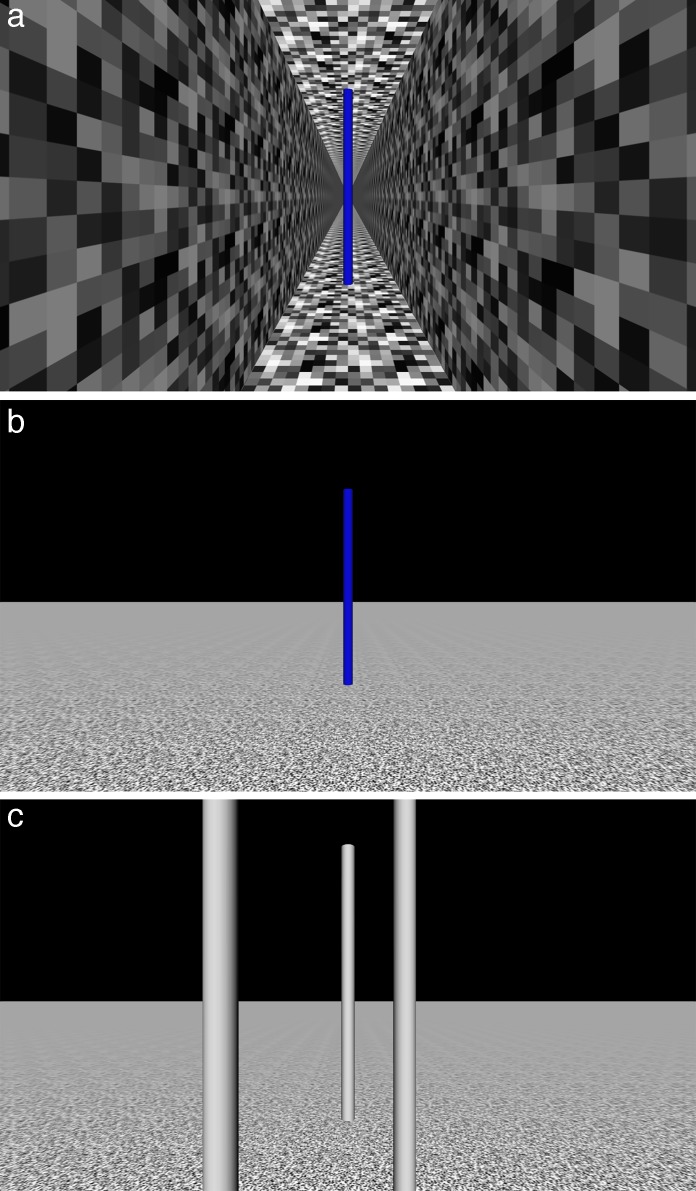
Examples of the virtual environments containing (blue) target post. (a) narrow corridor; (b) open ground plane; (c) obstacle environment (target behind far obstacle).

In the second experiment, participants wore a HMD that displayed a real-time view of a virtual environment. We used two different environments: a narrow corridor and an open ground plane, with or without obstacles (Figure 3).

We included two hemifield conditions: one in which the occluded hemifield was defined relative to the head, and one in which it was defined relative to the trunk. To aid direct comparison to Experiment 1, we also included a monocular viewing condition.

### Methods

#### Participants

The same participants that completed Experiment 1 took part in the second (virtual environment) experiment.

#### Equipment and virtual environments

The virtual environment experiment was conducted in the same room as Experiment 1, using the same equipment. The only difference was that participants perceived themselves in a virtual environment rather than in the physical motion-capture laboratory.

Participants wore a lightweight HMD (Rift DK2, Oculus, Menlo Park, CA) connected to the laptop computer carried in the backpack. The HMD provided a stereoscopic view of the environment, updated appropriately when the motion tracker signaled a movement of the participant's head. Position information was obtained at 75 Hz (to match the refresh rate of the HMD) via a wireless connection to the motion-capture system. This arrangement allowed the participant to walk freely without any trailing wires.

Virtual environments were created using Vizard. The first was a textured corridor; the second was an open ground plane (see Figure 3). As in the first experiment, the simulated environments contained start and target markers 7 m apart.

Participants carried a response button in their dominant hand and were asked to press it as soon as possible when, at random intervals of 1–3 s, the target marker changed color from blue to red. This task was introduced as a distractor task, in an attempt to reduce explicit attention to the walking path (for a similar design, see Saunders & Durgin, 2011). Although participants knew that their walking was being recorded, emphasis was placed upon the button-pressing task, leading the participants to concentrate on this rather than foot placement.

The three virtual environments used in the study were a corridor 200 m long, 2 m wide, and 4 m high (Figure 3a); an open ground plane 200 × 200 m in size (Figure 3b); and an open ground plane 200 × 200 m in size, containing three obstacles that participants were instructed to walk around (Figure 3c) to reach the target. The ground plane and walls were coarsely textured with a noise pattern to provide rich optic flow while walking.

**Figure 4:**
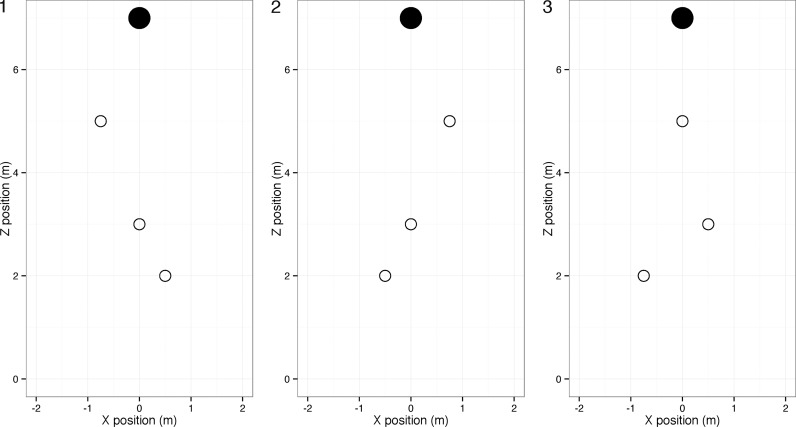
Plan view of the three obstacle (empty disk) layouts used in the study. Participants started at (0, 0) and ended at (0, 7).

Each of the three trials of each obstacle condition used a unique obstacle layout. The three layouts are shown in Figure 4.

#### Viewing conditions

The conditions were normal viewing (i.e., binocular, full field); left head-referenced hemifield loss; left trunk-referenced hemifield loss; and left-eye monocular loss. In the head-referenced condition, the left half of each display was extinguished. In the trunk-referenced condition, the visual field to the left of the trunk midline was extinguished (this was possible because we monitored both the trunk and head position). In the monocular condition, the left eye display of the HMD was extinguished.

As in Experiment 1, three walks were performed for each of the 12 conditions (4 viewing conditions × 3 environments).

#### Procedure

Walking trials in Experiment 2 took a similar format to those in Experiment 1, except for the use of the virtual environment. First the HMD was fitted and the participant was given the chance to become comfortable walking while wearing it. After participants reported being ready to begin, the trials commenced. Participants were required to begin each trial facing away from the target, before turning and walking toward the target marker at a regular pace set by the metronome. As before, they always walked in the same direction across the lab.

The order of conditions was counterbalanced using a Latin-square design; the three walks for each condition were performed together in a block. Due to a programming error, up to two of the final trials were omitted in the first six participants. However, due to the counterbalanced Latin-square design, no bias should have been introduced by these omissions.

To confirm that participants were not adversely affected by simulator sickness, they were asked to report any symptoms of nausea during the course of the experiment (and were again asked afterward). Had a participant reported feelings of nausea, the experiment would have been terminated. No participants reported feeling nauseous or disoriented at any time.

### Results

**Figure 5:**
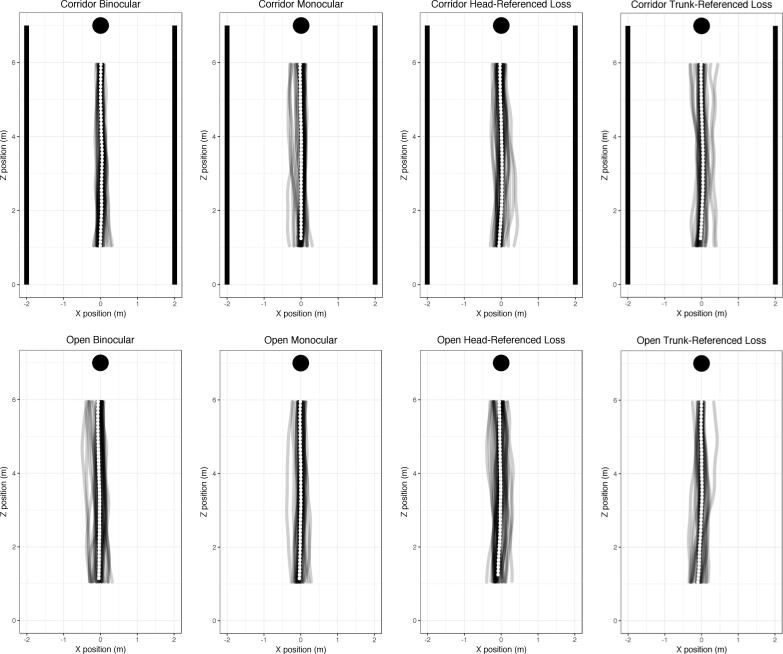
Walk trajectories in plan view. Participants started at (0, 0) and walked toward the target at (0, 7). Individual recordings are shown in gray, with the mean walk path (with error bars indicating standard error) overlaid in white for each 10-cm segment. Binocular, monocular, head-referenced, and trunk-referenced conditions are shown in four columns.

Walk-path trajectories for all participants in each condition without obstacles are shown in Figure 5. For each condition, the mean walk path (across individuals) is shown in white. The left column shows the baseline data—the trajectories taken under normal binocular viewing.

Table 1 compares the target-heading angle recorded under each modified viewing condition in each environment, as compared to binocular viewing conditions. The mean shift in target-heading angle across all monocular trials was 0.23° to the right, relative to trials with binocular viewing. Hedges's *g* was 0.16 (95% CI [−0.69, 1.01]).

**Table 1:**
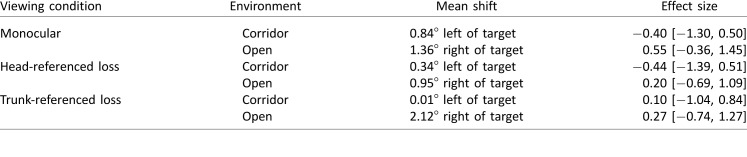
Comparison of the target-heading angle under each viewing condition and environment to binocular viewing conditions. Mean shift indicates the extent to which the visual manipulation affected walking toward the target. Effect size is Hedges's g, given with 95% confidence interval.

None of the differences reached clinical or statistical significance. Note, however, that a pattern is apparent—in the corridor and open environments, the paths curved in different directions. Using a post hoc ANOVA investigating environment and viewing condition, the main effect of environment did not reach statistical significance, *F*(1, 6) = 3.55, *η*_p_^2^ = 0.37, *p* = 0.11. Although the difference between the corridor and open environments did not reach significance, it prompted us to look for potential differences between the environments.

We investigated head turn (yaw). It has been reported that some people with HH may change their head posture to turn toward the blind field (particularly when HH is acquired early in life; for more detail, see Paysse & Coats, 1997). It has been proposed that these head turns may represent a strategy to maximize the “effective” visual field during exploratory saccades. When the head is turned, perceived straight-ahead is biased in the direction of the head turn (Howard & Anstis, 1974). Therefore, a difference in head posture between the two conditions would predict a difference in trajectories between the two conditions. Table 2 compares head yaw (with respect to the body) seen under each modified viewing condition in each environment, as compared to binocular viewing conditions.

**Table 2:**
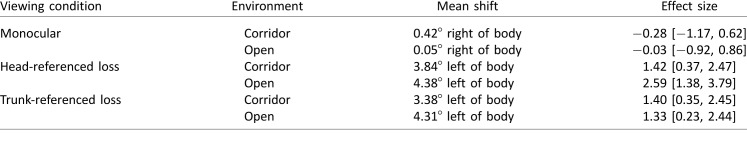
Comparison of head yaw under each viewing condition and environment to binocular viewing conditions. Mean shift indicates the extent to which the visual manipulation affected head yaw. Effect size is Hedges's g, given with 95% confidence interval.

Although there is a systematic difference in head yaw between conditions, it is immediately apparent that the head yaw (Table 2) does not match up with the target-heading angle (Table 1). The corridor-right/open-left pattern in the target-heading data is not found in the head-yaw data. Therefore, we can dismiss head yaw as a simple explanation for the small (and clinically and statistically nonsignificant) difference in curvature between the corridor and open conditions.

#### Obstacles

**Figure 6:**
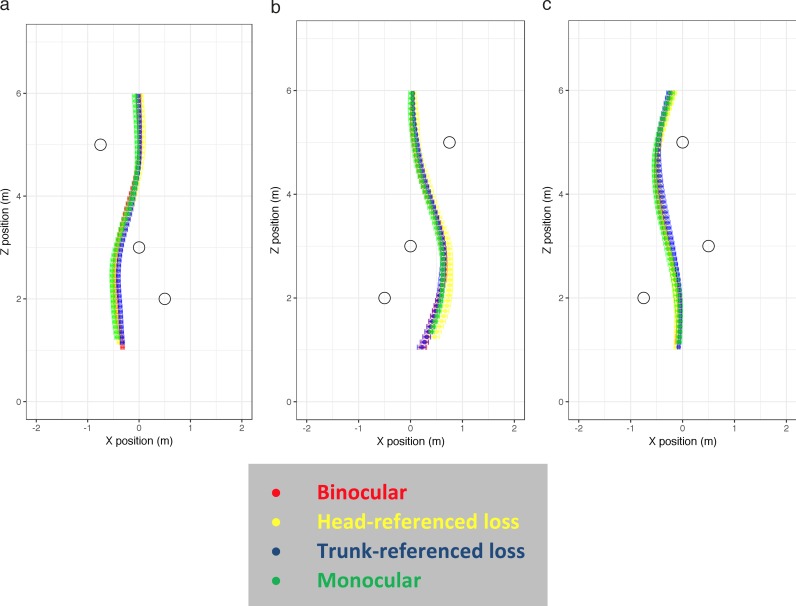
Examples of walk paths (in plan view) taken in each obstacle condition. Participants started at (0, 0) and walked toward the target at (0, 7). The most common walk path for each obstacle set is shown, with different viewing conditions overlaid. Error bars show the standard error. In obstacle condition 1 (a), two different routes were taken; the route shown was taken on 95% of the trials. In obstacle condition 2 (b), four different routes were taken; the route shown was taken on 68% of the trials. In obstacle condition 3 (c), four different routes were taken; the route shown was taken on 80% of the trials. For similar plots showing other chosen routes, see the Appendix.

In the obstacle trials, a variety of different routes were taken, but viewing conditions had no systematic effect on the route chosen (see the Appendix). For each obstacle arrangement, we identified the most commonly taken route between the obstacles (those shown in Figure 6) and used these for the statistical analyses. It is apparent that the trajectories are similar across conditions.

To quantify obstacle-passing distance, for each participant and each condition we first calculated the mean distance at which participants passed obstacles on each side. From these distances, we calculated the ratio of the left to the right passing distance, a measure of the bias introduced by the visual field restriction. We also calculated the grand mean passing distance, which we took as a measure of how confident participants were in each condition.

To confirm that it was valid to combine the data from different obstacle arrangements, we performed a 3 × 4 repeated-measures ANOVA and looked for significant interactions between obstacle arrangement and viewing condition. None were found—mean obstacle distance: *F*(6, 36) = 1.31, *η*_p_^2^ = 0.18, *p* = 0.28; left/right distance ratio: *F*(6, 18) = 0.60, *η*_p_^2^ = 0.17, *p* = 0.73.

Table 3 compares the left/right distance ratio seen under each modified viewing condition in each environment, as compared to binocular viewing conditions. Table 4 shows the effect on mean obstacle distance. None of the differences between viewing conditions reached clinical or statistical significance.

**Table 3:**
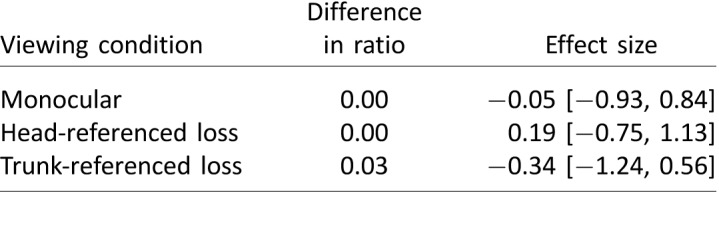
Comparison of the left/right obstacle passing-distance ratio under each viewing condition, as compared to binocular viewing. The difference in the ratio indicates, to two decimal places, the effect of the visual manipulation on the ratio. Positive values indicate that the participant passes closer to the obstacle on the right. Effect size is Hedges's g, given with 95% confidence interval.

**Table 4:**
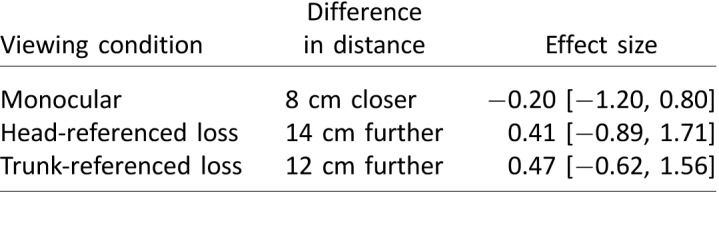
Comparison of the mean obstacle distance under each viewing condition, as compared to binocular viewing. The difference in distance indicates the effect of the visual manipulation on the mean distance participants were from obstacles when passing. Effect size is Hedges's g, given with 95% confidence interval.

### Discussion

This study, using a wide range of manipulations in virtual and real environments, investigated whether lateralized visual occlusion changes walking trajectory. None of the manipulations produced a statistically or clinically significant change in trajectory.

#### Hemianopia and unilateral visual neglect

People with restricted visual fields due to HH or UVN have difficulties walking. Our experiments were designed to try to tease out any role for a lateralized loss of vision or attention; we isolated lateralized field loss by simulating it in young healthy participants. Extrapolating from our findings with healthy individuals free of any deficits associated with brain injury, we conclude that it is unlikely that lateralized vision loss by itself explains the walking trajectories of people with HH and UVN.

It is important to note that although the visual manipulations used in this study represented our best efforts to simulate the visual consequences of HH and UVN using the available technology, there are important differences which may qualify our conclusion.

We occluded vision relative to the head. When the eyes are in the primary position (straight in the head), the effect on the visual field is the same as in occlusion relative to the eye (as in HH). We designed the task to encourage participants to keep the eyes in the primary position; they had to monitor a target placed directly ahead in the environment. However, this did not abolish all rotations of the head. We can estimate the extent to which a participant's gaze deviated from the primary position: Assuming the participant fixes their gaze on the target (as required by the task), the typical deviation of the eye from the primary position is simply the mean unsigned deviation of the head yaw from the target. This was 4.66° ± 1.44° across all participants in obstacle-free trials under head-referenced visual-occlusion conditions. During deviation of the head from straight-ahead, participants could see more or less of the scene on the occluded side of the target. However, because there is natural variability in what people with HH can see on the blind side of a fixated object—as some people have macular sparing (residual central vision in the blind hemifield), and eccentric fixation is also common (Hutchins & Corbett, 1997; Rowe, 2006; Trauzettel-Klosinski, 1997)—in practice this may have made little difference.

There are a number of important differences between our trunk-referenced condition and UVN. First, UVN is described as a gradient of attention or awareness running from the neglected to the nonneglected side of space (De Renzi et al., 1989; Kinsbourne, 1993), rather than a hard division between the two sides. In this respect, our trunk-referenced condition is effectively a more extreme version of UVN. Second, UVN is not just a neglect of one side of space defined relative to the trunk, but typically also a neglect of one side of objects (Driver & Mattingley, 1998). This is not something we attempted to simulate. However, when we consider the environments used (Figure 3), it is not obvious that it would make much difference apart from removing the left side of the posts in the obstacle environment. Third, there is some evidence that UVN is not solely tied to the trunk but includes a contribution of a retinal frame of reference (Mozer, 2002). Therefore, awareness of the scene should be modulated in part by where people look. We did not model this component of UVN. In practice, the eye, head, and trunk were approximately aligned most of the time due to the nature of the task—walk to a target straight ahead and maintain fixation on it. The most important difference between our simulation and UVN is that most people are not aware of their UVN (Kerkhoff & Schindler, 1997). This is clearly not something that can be simulated (and we note that previous attempts at simulating UVN have faced the same problem, e.g., Baheux, Yoshizawa, & Yoshida, 2007), so it is an important caveat when interpreting the results.

The experiments were motivated by an interest in the difficulties during walking that can be experienced following brain injury. For the reasons already outlined, our experiments should not be seen as direct simulations of UVN and HH. Such simulations are likely impossible, except potentially in the future by use of temporary perturbations of brain function such as those produced by transcranial magnetic stimulation. Our aim was to isolate and examine the consequences of a lateralized visual field loss on walking trajectories. Our results indicate that a lateralized loss of visual field does not on its own explain the difficulties encountered.

#### Attention during walking

In any experiment that involves walking in a laboratory, it is important to consider attention. Few experiments manage to recreate the normal state of mind wandering that characterizes the context for natural action (Schooler et al., 2011), and in most walking experiments participants pay an atypical amount of attention to the task of walking. In the introduction of Experiment 2, we explained the motivation for adding a distractor task for the participants to perform—we did not wish them to pay undue attention to walking. We believe that our distractor-task design achieved the primary aim of removing direct attention from walking (for a similar design, see Saunders & Durgin, 2011), but we were naturally unable to induce a more typical state of mind wandering.

#### Understanding the visual guidance of walking

We outlined three lines of research that lead to the prediction that visual-field loss would lead to changes in walking trajectory. What are the implications of the results reported here? The assumption that perceived egocentric directions would be biased was derived from the work of Porac and Coren (1986). The effect they reported was small but reliable (their results were based on data from 70 participants); with the left eye patched, they found a shift of approximately 0.35° to the right compared to binocular viewing. This is in agreement with the change we found in the overall mean target-heading angle (0.46° to the right in Experiment 1 and 0.23° to the right in Experiment 2), but the relationship does not hold when we separate open and corridor environments. It would be interesting to replicate their work to see if the shift in perceived direction differs between open and corridor environments, and if so, whether the difference maps onto the difference in walking direction we observed here.

Although there is evidence that humans are influenced by unequal flow rates on the left and right (Duchon & Warren, 2002), the effect is relatively weak and specific—it has been observed when participants walked between two virtual vertical planes (a virtual corridor with no floor). The effect was attenuated when a ground plane was introduced that intersected the bottom of the virtual walls and added perspective splay cues (Beall & Loomis, 1996). The lack of a significant difference due to occlusion in the corridor trajectories underlines how weak any unequal-flow effect is.

The bias in perceived heading due to an asymmetric flow field reported by Telford and Howard (1996) was fairly large (≥10°) when the head was fixed, but it was abolished when observers could rotate their head in the direction of locomotion. In this task, observers were able to directly view the target as they walked straight toward it. Fixation was consequently aligned with the direction of locomotion. The results here highlight the lack of ecological relevance of the asymmetry effect for understanding the visual guidance of walking with natural free fixation.

## Conclusions

The results indicate that the walking difficulties experienced by people with brain injury, specifically HH and UVN, are unlikely to be due to lateralized loss of vision or awareness on its own.

## Figures and Tables

**Figure A1:**
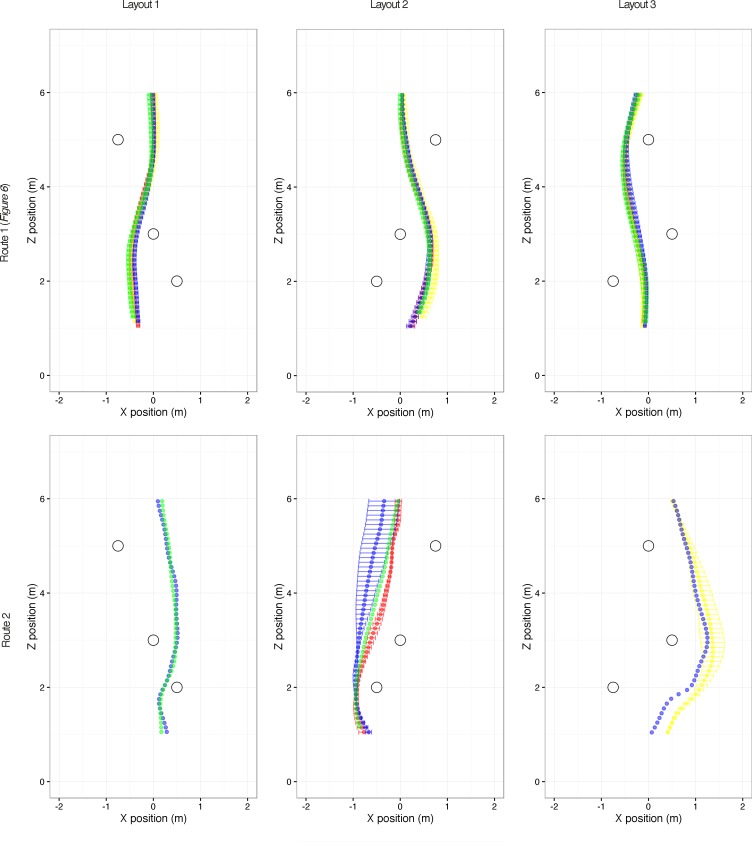
All routes taken (excluding trials with collisions, defined as the head passing within 20 cm of an obstacle) in the obstacle-avoidance task. Walk trajectories in plan view; participants started at (0, 0) and walked toward the target at (0, 7).

**Figure A1:**
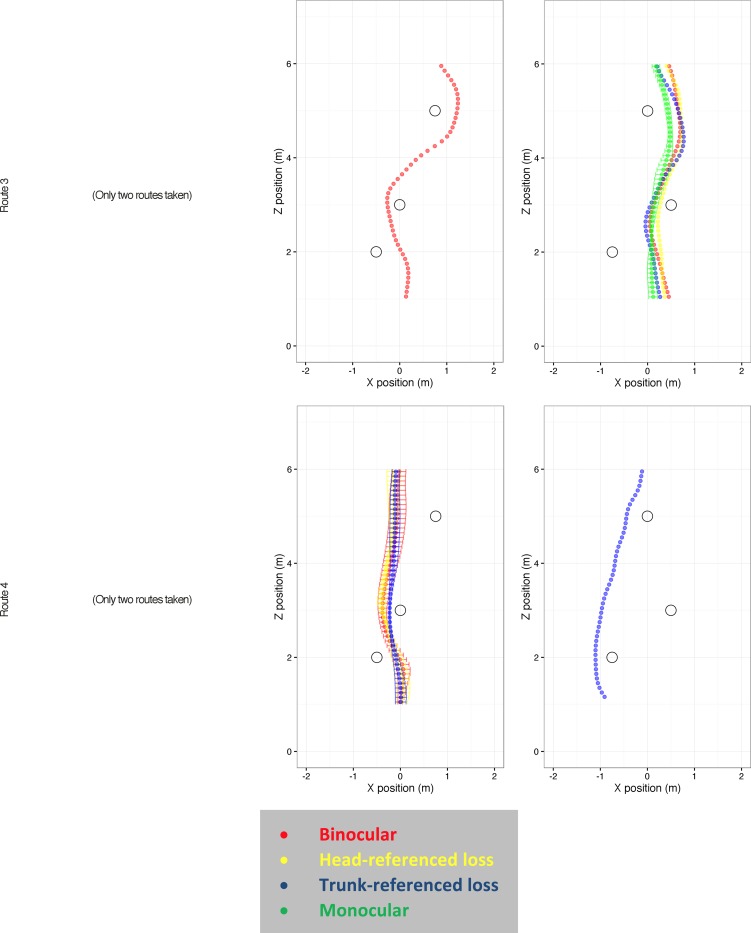
Continued.
